# Comparative genomics of Japanese encephalitis virus shows low rates of recombination and a small subset of codon positions under episodic diversifying selection

**DOI:** 10.1371/journal.pntd.0011459

**Published:** 2024-01-31

**Authors:** Mark Sistrom, Hannah Andrews, Danielle L. Edwards

**Affiliations:** 1 Department of Industry, Trade and Tourism, Berrimah Veterinary Laboratories, Darwin, Australia; 2 Research Institute for the Environment and Livelihoods, Faculty of Science and Technology, Charles Darwin University, Casuarina, Australia; 3 Department of Natural Sciences, Museum and Art Gallery of the Northern Territory, Darwin, Australia; NIAID Integrated Research Facility, UNITED STATES

## Abstract

*Orthoflavivirus japonicum* (JEV) is the dominant cause of viral encephalitis in the Asian region with 100,000 cases and 25,000 deaths reported annually. The genome is comprised of a single polyprotein that encodes three structural and seven non-structural proteins. We collated a dataset of 349 complete genomes from a number of public databases, and analysed the data for recombination, evolutionary selection and phylogenetic structure. There are low rates of recombination in JEV, subsequently recombination is not a major evolutionary force shaping JEV. We found a strong overall signal of purifying selection in the genome, which is the main force affecting the evolutionary dynamics in JEV. There are also a small number of genomic sites under episodic diversifying selection, especially in the envelope protein and non-structural proteins 3 and 5. Overall, these results support previous analyses of JEV evolutionary genomics and provide additional insight into the evolutionary processes shaping the distribution and adaptation of this important pathogenic arbovirus.

## Author summary

This comparative study of Japanese Encephalitis Virus is the largest genomic analysis of the virus to date. We undertake a suite of analyses to investigate phylogenetic relationships, rates of recombination and patterns of genomic selection. We show that recombination is not a significant driver of evolution in JEV, demonstrate support for previous phylogenetic reconstructions of the virus, and find a number of sites across the genome under episodic diversifying selection. These adaptive hotspots of evolution serve as key genomic points for the adaptive evolution of this important vector borne pathogen.

## Introduction

*Orthoflavivirus japonicum* (JEV) is an arbovirus belonging to the Flaviviridiae family with a zoonotic cycle involving swine as reservoir hosts, waterbirds as carriers and mosquitoes of the two genera *Culex* and *Aedes* as vectors [1,2]. While humans are dead-end hosts for JEV as they generally display low viremias insufficient to allow for infection of feeding mosquitoes [3], JEV infections of humans have significant health implications, with around 100,000 symptomatic cases of human JEV annually [4,5] resulting in approximately 25,000 deaths [6]. Only between 0.1–4% of human infections result in symptoms [7], however symptomatic cases have a fatality rate of 20–30% [8] and 30–50% of survivors develop long term neurological/psychiatric sequelae [9]. Despite the existence and use of several safe and effective JEV vaccines [8], JEV remains the dominant cause of viral encephalitis in the Asian region–meaning that understanding the evolutionary driving forces governing the range and pathogenicity of JEV are critical to ongoing management and control of this neglected tropical disease.

The JEV genome is 11kb, positive sense, single stranded RNA that comprises a single open reading frame encoding a large polyprotein that is co- and post-translationally cleaved into three structural proteins–capsid (C), precursor to membrane (prM) and envelope proteins (E) and seven non-structural, accessory proteins (NS1, NS2A, NS2B, NS3, NS4A, NS4B, NS5) [10,11]. The NS1 protein plays an essential role in genome replication [12]–mutations within this gene can have marked effects on RNA replication and infectious virus production [13]. NS2a and b are small, membrane associated proteins that play a role in virus assembly, RNA replication and interferon inhibition [14,15]. Mutations in NS2a have been shown to block virus assembly [16]. NS3 is a large, multifunction protein, encoding enzymatic activities necessary for polyprotein processing, RNA replication, virus assembly and apoptosis [16–18]. NS4a and b are both small, hydrophobic proteins. NS4a is involved in RNA replication via a genetic interaction with NS1 [19], and can induce membrane rearrangements and/or the formation of autophagosomes [20]. Mutations in NS4a confer resistance to flavivirus RNA replication inhibitors [21]. NS4b co-localizes with NS3 at sites of RNA replication, and is involved in blocking interferon signalling [22]. NS5 is large multifunctional protein involved in RNA capping and RdRP activities, as well as the induction of interleukin-8 secretion and blocking interferon signalling [23,24].

There are 5 recognized evolutionary lineages of JEV (GI-V) [25]. Historically, GIII was the dominantly detected strain, however it has recently been superseded by GI [25–27]. GII is largely confined to Southeast Asia and Northern Australia [28], and GIV and GV are generally confined to tropical Southeast Asia [29], however the 2022 outbreak of JEV in Australia was determined to be GIV, representing a range expansion of this genotype [30]. Vaccines are largely derived from GIII genotypes [29,31] and a growing body of evidence suggests that these vaccines show reduced efficacy toward GI and GV strains [32–34].

JEV is an evolutionarily dynamic pathogen with fluid transmission parameters associated with variations in host/vector range and climate change [35,36]. Further, virulence, pathogenicity and immunogenicity appear to vary between strains and remain in flux as the virus evolves in the face of exceptionally dynamic environmental factors [26,35]. Resultantly, contemporary comparative genomic studies are necessary to better understand and predict epidemiological patterns of JEV.

In this study, we analyse the complete genomes of 349 JEV isolates, identifying patterns of polymorphism, phylogeny, recombination and selection. We find that the genome is predominately under purifying selection; however, there are several sites which are subject to adaptive evolution across the phylogeny of JEV. It is likely that the adaptive evolutionary processes underlying the observed dynamism in the host, vector and geographic range of JEV, along with changes in virulence, pathogenicity and immunogenicity are being driven by a relatively small number of mutational changes at the genome scale.

## Materials and methods

We downloaded seven isolate genomes from the NCBI Short Read Archive (SRA) using SRAtoolkit v3.0.0 [37] and one assembly from the NBCI Assembly database. A further 356 complete genomes were downloaded from the NCBI Nucleotide database using Batch Entrez [38]. A further 36 samples were retrieved from the DNA Databank of Japan (DDBJ) SRA database [39], and a further 356 complete genomes were downloaded from the DDBJ nucleotide database [39], for a total dataset of 756 records. When filtered for redundancy (i.e. duplicate submissions in different databases), record accuracy (i.e. non-JEV samples entered in error) and length (i.e. minimum sequence length >10kb), a sequence set of 349 sequences were selected for further analysis (S1 Table). SRA genomes were filtered for read quality using Trimmomatic v0.32 [40] under default parameters, and aligned to a serotype O reference strain (GCA_000863325.1) using BWA v0.7.17 [41] with the mem function. Output SAM files were then sorted and converted to BAM format using SAMtools v1.17 [42]. Variant detection of each BAM file was undertaken using the mpileup function of BCFtools v1.17 [42] before being exported in FASTA format using the consensus function. SRA, Assembly and nucleotide data were then aligned in FASTA format using MUSCLE v5 [43] using the super5 algorithm and guide tree permutation enabled.

Recombination across the genome was calculated using the program RDPv5 [44] with default settings, which implements several methods to detect recombination in a given sequence alignment. Phylogenetic reconstructions were undertaken for the whole genome, as recombination was not found to have a significant impact on genome structure. Phylogenetic reconstructions were conducted using RaxML [45] with default settings and 1,000 bootstrap replicates, using Murray Valley encephalitis virus (MDV) as an outgroup. Phylogenetic model selection was conducted using the Bayesian information criterion implemented in jModelTest2 [46]. Trees were estimated using a GTR model. Selection was initially evaluated for the whole genome by calculating nucleotide diversity (π, the average pairwise difference between all pairs of sequences) and (θ, the Watterson’s estimator of nucleotide diversity) using a sliding window analysis with a window length of 100 and step size of 25 sites implemented in DNAsP v6 [47]. Selection for each gene was tested initially using a codon-based Z test of neutrality implemented in MEGA11 [48]. We calculated overall average Dn-Ds for each gene and probability of neutral model fit using the Nei-Gojobori method with 500 bootstrap replicates. Missing sites were treated with partial deletion with a cut-off of 95%. We further evaluated selection using a number of analyses implemented in the HyPhy2.5 [49]. We implemented a Branch-site Unrestricted Statistical Test for Episodic Selection (BUSTED) [50] to evaluate each gene for episodic selection using the previously calculated tree along all branches of the phylogeny. Secondly, a Mixed Effects Model of Evolution (MEME) [51] was used to test each gene for specific sites under diversifying selection using the same tree as a guide. Finally, we conducted an adaptive branch-site random effects model for episodic selection (aBSREL) analysis on phylogenies for each gene independently to detect branches under episodic selection.

## Results and discussion

### Recombination

**Fig 1:**
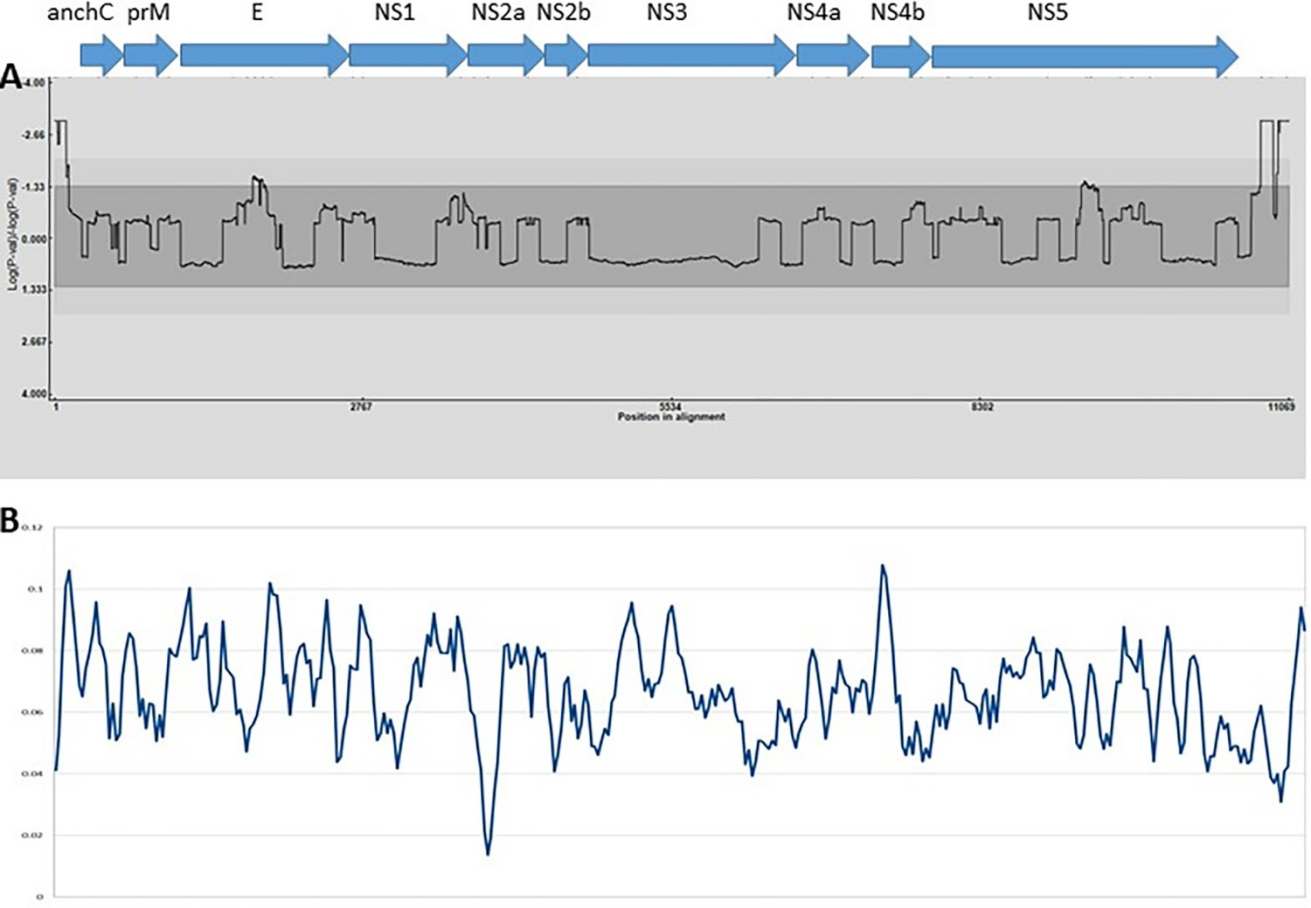
Recombination and selection analysis of JEV genomes. Top legend indicates the locations of protein coding genes, bottom legend indicates position in the genome alignment. Graph A depicts recombination break point probability per site, light grey indicates 99% probability, the mid grey area indicates 95% probability while the dark grey represents non-significant break points. Marginally significant break points were observed in structural protein E and Nonstructural protein 5. Graph B depicts nucleotide diversity (π) calculated using a sliding window analysis.

**Fig 2:**
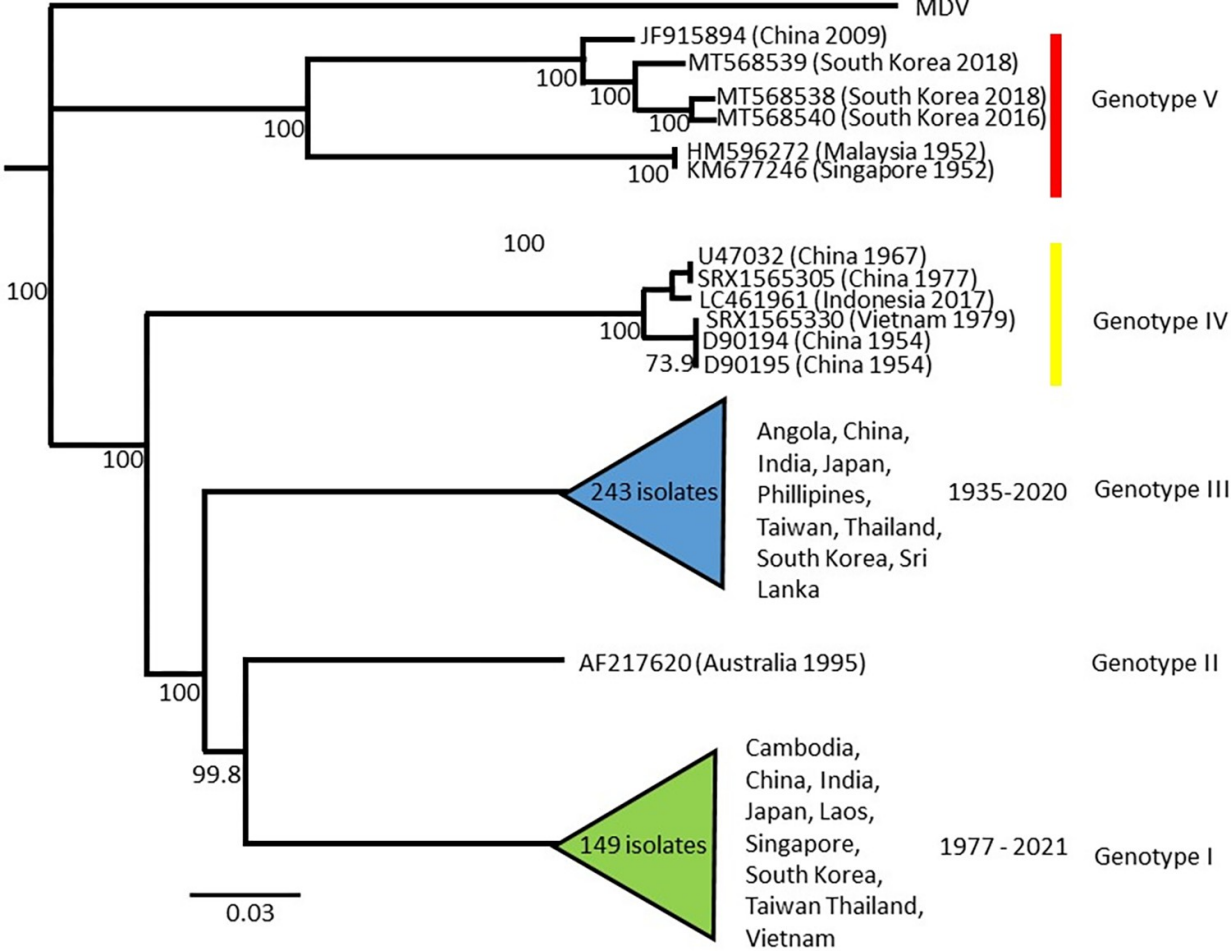
Phylogeny of JEV genome sequences, showing the five monophyletic genotypes of JEV. Branching order in the current analysis is confirmatory of prior studies of evolution of JEV. Node labels indicate bootstrap support, scale is in substitutions per site. The MDV sequence is a Murray Valley encephalitis virus sequence used as an outgroup for the phylogenetic analysis.

The results of recombination analyses are reported in Table 1 and Fig 1. We found limited evidence of recombination in our dataset, especially in comparison with other viruses for which recombination is noted to be a driving evolutionary force [52–54]. Recombination is not widely reported in unsegmented arboviruses [55], however recombination via template switching is thought to be an important driver of evolution in RNA viruses [56] and thought to be most likely to occur within vertebrate hosts during multi-strain infections [57]. Recombination of JEV genotypes would have significant potential implications for vaccine efficacy, as vaccines derived from GIII isolates show reduced efficacy in toward GI and GV strains [32–34]. We did find evidence of a small number (n = 18) of recombinant strains from both GI and GIII isolates, largely between closely phylogenetically related isolates and in the mid region of the E protein and NS5 protein respectively (Table 1 and Fig 2). One instance of recombination between a GI and GIII strain was detected, but the support for this recombination event was found only by two of five methods implemented by RDP5 [44] and comprised of a short fragment in the 3’ end of the genome, which contains the most missing data in our alignment. Resultantly, this result is low confidence. We therefore conclude that recombination during multi-strain infection is unlikely to be a major driver in the evolution of genomic diversity in JEV.

**Table 1:** Recombinant strains as detected by RDP5 analysis. All events were between isolates of the same genotype with the exception of those with unknown parents, and event 16. R–RDP, G–GENECONV, B–Bootscan, M–MaxChi, C–Chimaera, S–SiScan, T– 3Seq, Major parent–major presumed donor of recombined sequence, Minor parent–minor presumed donor of recombined sequence (if more than one recombination event detected).

Event	Recombinant Isolate	Genotype	Major Parent	Genotype	Minor Parent	Genotype	Detection Method						
							R	G	B	M	C	S	T
1	MH385014	I	JN381831	I	AY303798	I	+	+		+	+	+	+
2	AF045551	I	GU187972	I	AY303798	I	+	+	+	+	+	+	-
3	KU351668	I	unknown				+	+	+	+	+	-	+
4	GU187972	I	unknown				-	+	-	-	-	-	+
5	JF706282	I	JN381851	I	KT957423	I	+	+	+	+	+	+	+
6	MH753128	III	AF069076	III	NC_001437	I	+	+	+	+	+	-	+
7	KU351667	I	KU508408	I			+	+	+	+	+	+	+
8	KY885009	III	AF069076	III			+	+	+	+	+	+	+
9	JN381834	I	JN381851	I			+	+	-	+	+	+	+
10	AF098737	III	JEVBEICG	III	unknown		+	+	+	-	-	-	+
11	JN381868	III	JF706273	III			+	+	-	+	+	+	+
12	KU871322	III	KU871363	III			+	+	+	-	-	-	+
13	JF706269	III	JEVBEICG	III			+	+	+	-	-	-	+
14	JN381864	III	JN381865	III			-	-	-	+	+	+	+
15	KU508409	I	MZ540901	I			+	+	+	-	-	-	+
16	MZ702743	III	KT229572	I			-	-	-	+	+	-	-
17	JN381866	III	JN381858	III			+	+		+	+	-	+
18	AF098736	III	unknown		JEVBEICG	III	+	-	-	+	-	+	-

### Phylogenetic relationships

Our phylogenetic analysis resolved the five previously identified genotypes of JEV with high confidence (Fig 1). As we did not identify any novel genotypes in our data, this analysis is largely confirmatory of prior studies [10,26,35] as we found the JEV genotypes fell into 5 reciprocally monophyletic clades in an ascending pattern of diversification from V–I (Fig 1). While we find support for the current classifications of Genotypes I–IV, there is a deep divergence between isolate JF915894 (China, mosquito, 2009) and HM596272 (Malaysia, human, 1952)/KM677246 (Singapore, human, 1952), which may warrant further classification of this genotype (i.e., Genotype V), pending sequencing of additional isolates. We also find two clades within Genotype I conforming to the previously identified clades 1a and 1b [58], however bootstrap support in our analysis was low (56%).It is notable that Genotypes II, IV and V are represented by only one, six and six isolates respectively despite the significant increase in size of the present dataset, indicating either a low prevalence of these genotypes in nature, or a considerable bias in the collection and public dissemination of JEV genomic data. The recent increase in the circulation of Genotype V following 57 years of apparent extinction [59] and expansions into novel geographic areas [60] is an important evolutionary and epidemiological development. Additional genome sequences of this genotype will be critical for determining the evolutionary adaptive context and epidemiological implications of this expansion, especially in the face of potentially reduced efficacy of current vaccines on Genotype V infections [32–34]. It is noteworthy that the present study is based on full genomes and that a gene specific approach may increase sampling density of these genotypes.

### Selection

Nucleotide diversity analysis showed relatively low rates of mutation across the genome (Fig 2, Graph B), with a notable decrease in π in NS2a and a notable increase in π in NS5. These rates of nucleotide diversity did not necessarily correspond with complementary changes in Dn-Ds ratios (Table 2). All genes deviated significantly from a model of neutral evolution, with strongly negative Dn-Ds values indicative of a strong overall signal of purifying selection.

**Table 2:** Results of selection analysis. Test of neutrality was conducted using an averaged codon based, two tailed Z test; Episodic selection was determined using a Branch-site Unrestricted Statistical Test for Episodic Selection; and codon positions under diversifying selection were determined using a Mixed Effects Model of Evolution analysis. The most prevalent amino acid substitution is indicated after each position when non-synonymous.

Gene	Test of neutrality		Episodic Selection (P)	Positions Under Diversifying Selection	
	Dn-Ds	P			Branches Under Diversifying Selection
**AnchC**	-20.44	0.00	0.50	-	
**prM**	-10.6	0.00	0.17	143 (Gly > Arg)	KU351668 (Genotype III, China, 2008)
**E**	-19.73	0.00	0.00	34, 37, 49 (Glu > Lys), 51, 72 (Ala > Thr), 129, 135, 138 (Glu > Lys), 160 (Gly > Arg), 176 (Ile > Thr), 209 (Lys > Arg), 227 (Ser > Pro), 244, 264 (Gly > His), 280, 307, 310, 390, 434, 472	HM228921 (Genotype III, China, 1960)
**NS1**	-16.74	0.00	0.01	11 (Lys > Arg), 25, 75 (Arg > Phe), 94, 175 (Asn > Asp), 178 (Glu > Gly), 206 (Leu. Tyr), 215, 276	
**NS2a**	-11.54	0.00	0.00	4 (Glu > Gly), 88, 132	
**NS2b**	-10.13	0.00	0.03	36	
**NS3**	-22.57	0.00	0.00	13 (Cys > Ser), 28, 39 (Gly > Arg), 58, 68 (Tyr > His), 78 (Ala > Thr), 95, 96, 101, 102, 115 (Val > Ile), 150, 235, 251, 340, 341, 342, 343 (Ser > Arg), 344, 347 (Ser > Arg), 348, 350, 474, 482 (Asp > Asn), 528	LC461958 (Genotype I, China, 2006) AF221499 (Genotype III, Taiwan) AF2211500 (Genotype III, Taiwan, 1994)
**NS4a**	-11.41	0.00	0.31	-	
**NS4b**	-14.96	0.00	0.04	24, 45 (Leu > Phe), 49, 112, 133, 178, 199 (Ala > Arg)	
**NS5**	-26.19	0.00	0.00	15 Ala > Gly), 161, 205 (Trp > Gly), 270, 346 (Asp > Gly), 416 (Arg > Ala), 493, 505 (Leu > Ile), 512 (Pro > Glu)513, 580, 628, 672 (Asn > Thr), 677 (Arg > Lys), 752	GU187972 (Genotype III, China, 1950) AF098735 (Genotype III, India, 1978)

Branch-site Unrestricted Statistical Test for Episodic Selection (BUSTED) (50) analysis showed that some branches in the phylogenetic tree for the genes E, NS1, NS2a, NS2b, NS3, NS4b and NS5 all displayed episodic diversifying selection (Table 2). Within these genes, a Mixed Effects Model of Evolution (MEME) [51] showed that a small proportion of sites (0.7% of the genome) displayed episodic diversifying selection (Table 2) with the number of sites in each gene found to be under diversifying selection corresponding to the significance of the results of the BUSTED analysis. While summary statistic approaches demonstrate strong evolutionary conservation of genotypes at a genomic scale, more nuanced analysis shows that a small number of sites throughout the genome are diversifying in a manner likely to be adaptive. Of particular interest is the strong episodic diversifying selection of protein E–the major surface protein of the virion. Experimental evolutionary approaches have shown that specific mutations in surface proteins can generate thermo-tolerance in viruses [61,62] and the mutations observed in the JEV E protein may assist in the evolution of this virus to persist in novel environments.

Three mutations observed to be under selection in the E protein (i.e., residues 307, 310 and 390) fall within the β-barrel structure of the protein, thought to be involved in antigenic variation in JEV [63]. Further, seven of the mutations observed to be under selection fall in the Envelope Domain I (EDI) region (i.e., residues 34, 37, 49 (Glu >Lys), 51, 138 (Glu > Lys), 160 (Gly > Arg) and 176 (Ile > Thr)) which controls the orientation of the E protein [64]. The envelope protein mutation E138 detected in this study is a well-characterized neurovirulence associated mutation [65,66]. E176 and E264 also shows reduced neurovirulence in experimental studies [66], with E176 operating synergistically with E138 [66]. West Nile Virus with mutations in residues 67 and 153 of the EDI region show reduced cellular attachment and neuroinvasiveness [67,68], and the mutation at residue 390 lies in a neutralising epitope of Envelope Domain III region (EDIII) in which mutations have been shown to affect cell tropism and virulence [64], however the mutation detected under episodic selection is synonymous in our results.

The Pre-membrane protein (PrM) is important for viral maturation, and mutant variants of PrM have shown increased rates of replication [69], however the selective impacts of the mutation observed in this study (143 (Gly > Arg)) is unknown.

Mutations under selection observed in the NS2a gene fall within three transmembrane segments (pTMS), pTMS1 (4 (Glu > Gly)), pTMS3 and pTMS5 (132) [70]. pTMS1 does not have membrane associated activity, and it’s involvement in the virus life cycle is unclear, however NS2a mutants with substitutions in pTMS1 showed a >1,000-fold reduction in virus yield, an absence of plaque formation and infectious-virus-like particle yields [71] in dengue virus.

A mutation detected as under episodic selection in the NS2b gene (i.e. NS2b78) in this study was also experimentally demonstrated to impact replication (72).

NS3 is a multifunction protein, in which we observed a synonymous mutation at site 150 under selection, in which mutations have been shown to reduce protease activity in dengue virus [71]. In JEV the A78S mutation, which was observed to be under selection in our study, has an effect on viral replication *in vitro* [72]. A mutation we observed as under selection in this study in NS4b was also found to be under selection between genotypes I and III in a prior study [27].

NS5 is another large multifunctional protein in which we observed several mutations under selection. While several mutations in NS5 have been shown in in vitro studies to be adaptive in the NS5 gene in JEV [73,74], the mutations we observed to be under selection in this study were not among those seen to be experimentally validated, indicating that further experimentation is necessary to determine the causative nature of these mutations.

Similarly, large numbers of sites under diversifying selection were observed in the large, multifunction, non-structural proteins NS3 and NS5, but due to their multifunctional nature, the specific selection pressures driving the diversification of these mutations will be difficult to determine. The mutations under selection we observed in JEV warrant further investigation relevant to their virulence and infectivity.

Finally, we conducted an adaptive branch-site random effects model for episodic selection (aBSREL) analysis and detected branches under episodic selection in the E, NS3 and NS5 proteins, with one, three and two terminal branches under episodic diversifying selection respectively (Table 2). The genomes represented by these branches are either Genotype I or III, and were collected in either China or Taiwan. The dates of isolation range from 1950 to 2008. Given 99.7% of the genome is under stabilizing selection, it is expected that relatively few branches would display episodic diversifying selection, however it is somewhat novel that the branches that display this selection are not from Genotypes IV or V, which are the genotypes showing the most rapid geographic and epidemiological shifts in JEV.

As an analysis of publicly available sequence data submitted to various databases, the present study is inherently limited by the presumed accuracy of these sequences and the completeness of the metadata submitted with those sequences. It is also limited by the inherent biases in the isolates which are chosen for sequencing by the broader scientific community.

## Conclusions

Our study conforms to the results of previous studies to demonstrate the five distinct genotypes of JEV determined at the gene level are robust with increased sampling and whole genome phylogenetic analysis. We did however find a relatively deep divergence within Genotype V–albeit based on a small number of isolates. This potentially warrants further splitting of the recognized genotypes in JEV pursuant to the examination of additional isolates.

We demonstrate, like previous studies [25,75,76] that recombination is likely to occur at relatively small scales within JEV genotypes, and confirm a previously identified recombinant JEV strain (i.e. K94P05) [25,76]. Despite significant geographic co-occurrence of genotypes facilitating potential co-infection of hosts with multiple strains of JEV [58,77], it seems as though recombination is unlikely to be a major driver of genomic diversity and evolution.

We show widespread purifying selection acting on the JEV genome, consistent with previous analyses of selection in JEV [58,75] and other flaviruses [78]. It is thought that this strong purifying selection acting on the genome is because JEV relies on infection of both a vertebrate and an arthropod host, meaning that JEV has to endure at least two selective environments during its replication cycle [58]. Despite this overall pattern of purifying selection, we detected a number of sites under episodic diversifying selection. Experimental approaches to determine the functional impact of other mutations shown to be under episodic diversifying selection are likely to yield insights into the evolutionary driving forces that precipitate geographic range, host and vector expansion in JEV.

## Supporting information

S1 Table Metadata for genome sequences used in this study including Accession numbers, host, country, strain name and reported genotype. (XLSX)
